# *De-novo* reconstruction and identification of transcriptional gene regulatory network modules differentiating single-cell clusters

**DOI:** 10.1093/nargab/lqad018

**Published:** 2023-03-03

**Authors:** Mhaned Oubounyt, Maria L Elkjaer, Tanja Laske, Alexander G B Grønning, Marcus J Moeller, Jan Baumbach

**Affiliations:** Institute for Computational Systems Biology, University of Hamburg, Hamburg, Germany; Chair of Experimental Bioinformatics, TUM School of Life Sciences Weihenstephan, Technical University of Munich, Freising, Germany; Department of Neurology, Odense University Hospital, Odense, Denmark; Institute of Clinical Research, University of Southern Denmark, Odense, Denmark; Institute of Molecular Medicine, University of Southern Denmark, Odense, Denmark; Institute for Computational Systems Biology, University of Hamburg, Hamburg, Germany; Novo Nordisk Foundation Center for Basic Metabolic Research, Faculty of Health and Medical Sciences, University of Copenhagen, Copenhagen, Denmark; Heisenberg Chair of Preventive and Translational Nephrology, Department of Nephrology, Rheumatology and Clinical Immunology, RWTH Aachen University, Aachen, Germany; Institute for Computational Systems Biology, University of Hamburg, Hamburg, Germany; Department of Mathematics and Computer Science, University of Southern Denmark, Odense, Denmark

## Abstract

Single-cell RNA sequencing (scRNA-seq) technology provides an unprecedented opportunity to understand gene functions and interactions at single-cell resolution. While computational tools for scRNA-seq data analysis to decipher differential gene expression profiles and differential pathway expression exist, we still lack methods to learn differential regulatory disease mechanisms directly from the single-cell data. Here, we provide a new methodology, named DiNiro, to unravel such mechanisms *de novo* and report them as small, easily interpretable transcriptional regulatory network modules. We demonstrate that DiNiro is able to uncover novel, relevant, and deep mechanistic models that not just predict but explain differential cellular gene expression programs. DiNiro is available at https://exbio.wzw.tum.de/diniro/.

## Graphical Abstract

**Graphical Abstract:**
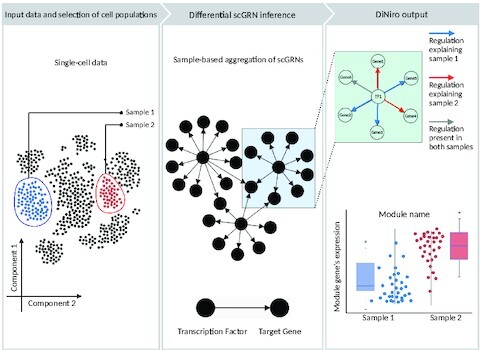
DiNiro a method for uncovering disease mechanisms at the single-cell level through small, interpretable transcriptional regulatory network modules.

## INTRODUCTION

In recent years, single-cell RNA sequencing (scRNA-seq) has been demonstrated as an emerging field that offers unprecedented opportunities to study cellular heterogeneity and gene expression variability at a high resolution. The power of single-cell sequencing technologies (Drop-seq (1), Fluidigm C1, Smart-seq2, 10X Genomics Chromium (10X; 10X Genomics, Pleasanton, CA)) to simultaneously assess the gene expression profiles of millions of cells within a sample (2,3) has already led to various novel and interesting discoveries (4,5). However, as single-cell technologies improve, our ability to generate scRNA-seq data exceeds our capacity to extract valuable information from it. Thus, there is a need to develop more advanced computational methods to conduct more analyses besides ameliorating the standard analysis (e.g. quality control, cell type identification, differential expression and trajectory inference) in order to gain as many biological insights as possible from the data (6). In this context, differential single-cell network enrichment analysis of scRNA-seq data holds enormous potential to reveal important and interesting findings but have not yet been fully explored.

In principle, a typical scRNA-seq analysis has two main components: pre-processing and downstream analysis (7). The pre-processing step includes, but is not limited to, the following: quality control, normalization, batch-effect correction, feature selection, and dimensionality reduction. The pre-processing steps aim to assess the biological soundness of the data and to weaken the effects of noise before downstream analysis. This latter can be categorized into cell- and gene-level downstream analysis. Gene-level analysis includes the study of co-expression, gene variability and differential expression. A wide range of tools and packages have been developed to facilitate this analysis-type and to mine biological information from the largely available scRNA-seq data (8–11). Taking scRNA-seq gene-level analysis one step further and performing differential network enrichment analysis can help discover regulatory phenotype or disease mechanisms, which is not possible with co-expression or differential expression analysis (12).

Differential co-expression analysis may identify gene pairs, i.e. transcription factors (TFs) and their target genes (TGs), also called modules, showing distinct co-expression patterns across cell clusters. For instance, a module may be co-expressed in one sample but not in another sample. Co-expression of genes can be modeled as a network, where genes are connected, when an appreciable co-expression association between them exists (13). One frequently used approach for co-expression analysis is Gene Regulatory Networks (GRNs) inference. GRNs are typically modelled as directed graphs with two types of nodes (TFs and TGs) and directed edges describing a regulatory interaction (activation or repression) between a TF and a TG (14,15). Efficient learning of GRNs from scRNA-seq data (scGRN) (8,16–21) is crucial for revealing the underlying molecular mechanisms governing cellular programs that drive phenotypic developments, such as diseases or cell and tissue types. Additionally, analyzing GRNs helps understanding disease progression, disease subclassification, and pinpointing biomarkers responsible for phenotypic differences (22). However, tackling this task remains challenging (23) due to a large amount of technical problems (e.g. library size differences (24), cell cycle effects (25), amplification bias, and low RNA capture rate (26)) and biological noise (e.g. stochastic transcription) embedded within scRNA-seq data that corrupt the biological signals and obstruct the analysis. On top of this, conducting this analysis at single-cell level raises new challenges, specifically the un-synchronization of TF dynamics and gene expression because of stochastic variation and transcriptional bursting (27–29), which affect capturing per-cell existing TF–TG correlations that are decisive for modeling the regulatory relations and constructing single-cell gene regulatory networks (scGRNs).

scGRNs construction from scRNA-seq data has limited success (30,31). Furthermore, benchmarking studies on both synthetic and real data have revealed that existing methods have limited accuracy. While tools such as PPCOR (32) and PIDC (33) use partial correlation and partial information decomposition to identify gene coexpression modules, respectively, few methods can identify directed networks. SCENIC (34), a tool for identifying directed networks, prunes edges based on known transcription factor binding sites. Other GRN inference approaches based on pseudotime analysis exist (35–37), but their performance is generally inferior to those based solely on scRNA-seq data. However, we still lack tools for differential scGRN reconstruction.

Here, we introduce **DiNiro**, the first tool for conjoint *de novo* differential scGRN reconstruction and network enrichment aimed at the identification of molecular regulatory mechanisms directly from scRNAseq data. DiNiro is implemented as an interactive web tool capable of capturing the variation in co-expression patterns across two conditions based on gene expression profiles. We use Copulas (38–40) to model the dependency between expression profiles of gene pairs (i.e. TF and a TG) in individual samples. Copulas are functions that allow estimating the joint distribution of the gene expression profiles in each sample as a function of marginal distributions. The distance between the in-sample copulas of a gene pair (TF, TG) signifies the difference in the regulation across samples. Given the noisy and sparse nature of the single-cell data, the scale-invariant property of copula (41) passed down to DiNiro makes the distance measure approximately scale-invariant, increasing its ability to detect minor changes in co-expression in gene pairs expression profiles derived from a chaotic unbalanced (i.e. the compared cell clusters are of different sizes) expression data. It can detect minor differences in correlation between two conditions, which is the most desired aspect of any differential coexpression analysis.

In the following, we first give an overview of the technological concept of DiNiro (extensive details are given in the Materials and Methods section). To illustrate the robustness and sensitivity of DiNiro, we assessed its performance on simulated data with known regulatory interactions. The results highlight the capacity of DiNiro in capturing the inter-sample induced biological regulatory variation. Furthermore, we analyzed different real-world datasets. Beside validating the findings of the original studies, DiNiro was also able to retrieve novel findings about the differential molecular interactions, which provided an extra layer of biological meaning to the scRNA-seq data.

To clarify terminology, throughout this paper, we will refer to subnetworks of connected genes as ‘modules’. We refer to a set of cells with the same phenotype as a ‘cluster’, and by contrast, a sample refers to a set of cells picked by the user either from cells of one cluster or more.

## MATERIALS AND METHODS

### DiNiro overview

**Figure 1.**
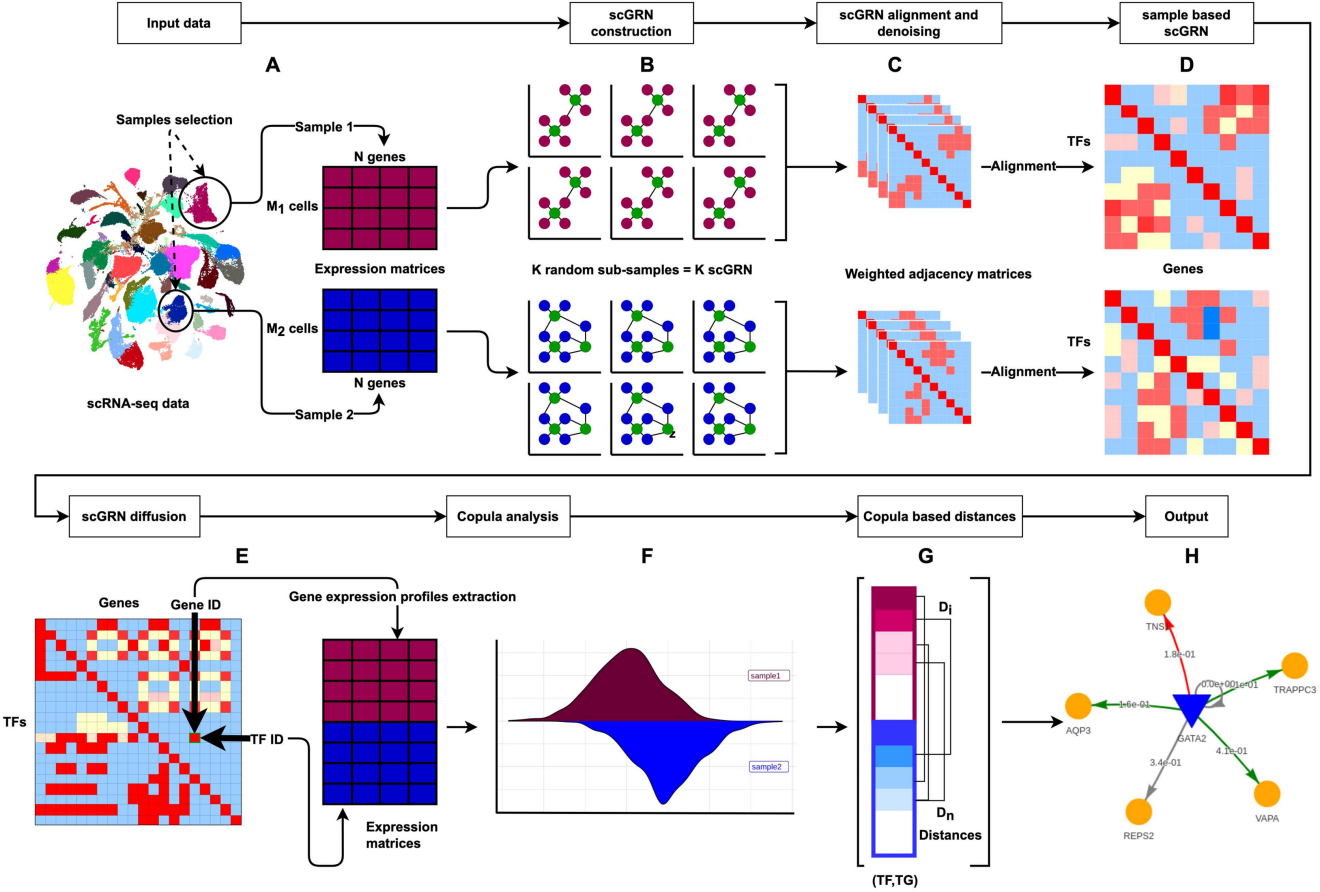
DiNiro workflow. (**A**) The analysis starts with selecting two samples from the input single-cell data. Each sample undergoes a random subsampling process. (**B**) The scGRNs are inferred from the sub-samples using GRNBoost2 to identify the potential gene target for every TF based on co-expression. RcisTarget is used to filter the false predictions based on a *cis-*regulatory TF binding motifs enrichment analysis. (**C**, **D**) subsampling-based scGRNs are denoised and aligned to construct one precise sample-based scGRN. (**E**, **F**) The two scGRNs are diffused into a TFs $ \times$ TGs adjacency matrix, and fed to the Copula-based model to identify differential co-expression modules. Empirical Copula is used to measure the dependence of each transcription factor–target gene (TF–TG) pair expression profile in every sample. (**G**) The distance between a TF–TG pair models the difference in the regulatory relationship between each regulator and its target across the two samples. (**H**) Example of an output module, where the color-coded edges indicate whether a gene pair is regulated in one sample and not in the other or in both.

DiNiro permits the comparison of user-chosen single-cell samples to unravel gene modules driving regulatory mechanisms underlying diseases or cellular programs that govern disease progression. DiNiro takes as input a single-cell gene expression matrix as an AnnData object in H5AD file format. The cell maps contained within the uploaded AnnData object are used to display the cells as color-coded points (e.g. UMAP or PCA as a map and cell type as a color). The user can interactively select two samples to be compared based on cell clusters or by drawing a freehand circle to select cells (i.e. Lasso selection) (Methods and Figure 1A). Upon sample selection, an scGRN is generated for each sample. To reconstruct this sample-based scGRN, DiNiro first sub-samples the cells and computes different scGRNs, which are then combined into one precise sample-based scGRN (Materials and Methods and Figure 1B–D). The reasoning behind this process is to assist in reducing signal-to-noise ratio and filter out false predictions. See Methods for a detailed explanation. DiNiro then performs a differential network enrichment analysis to identify meaningful changes in pairs (i.e. a TF and a TG) regulatory relationships across samples. The copula function is used to model the correlation of the pairs in each sample, and the copula distance represents the difference in the regulatory relationship of the pair across the two samples (Methods and Figure 1E–H). DiNiro offers a range of output including the differential modules and their statistically significant Gene Ontology (GO) terms. It also provides informative and intuitive visualizations of the resulting modules as networks with sample-based edge view navigation, a searchable Venn diagram of overlap and unique TF–TG interactions between samples, and tables with a list of genes regulated differently in each sample or both.

### Numerical methods

#### Input data and selecting samples

As a starting point, the single-cell data is loaded into DiNiro is an *M*$ \times$*N* expression matrix, where each row represents a cell, and every column represents a gene. Each entry in the matrix represents the expression level of a particular gene in a given cell. The inputted AnnData object (42) has also extra metadata describing all the pre-processing analysis (e.g. UMAP coordinates of each cell). Prior to sample selection, the user can select the plotting map and the coloring criteria to be used for the purpose of interacting with the data. To proceed with the analysis, at least two different samples (i.e. sample A and sample B, a sample can be formed from cells from different clusters) need to be selected, where a sample is an expression matrix consisting of cells from at least one cluster. Sample A an *M*_A_$ \times$*N* (A) and sample B an *M*_B_$ \times$*N* (B), *M*_A_$ \le$ *M* and *M*_B_$ \le$ *M*. After the subsampling step, the user needs to specify two parameters (the default parameters, and the effects of parameter settings on the analysis are given in Supplementary Materials S1): *K* number of sub-samples to acquire for each sample and Pct (%) a subsampling percentage. The subsampling process is carried out as follows: (*M*_A_ /100 × Pct) cells are randomly chosen from A, *K* times (i.e. same goes for B). This yields *K* submatrices for each sample noted as {a1, a2,…,a*k*} and {b1,b2,…,b*k*}, respectively.

We use an approach consisting of GRNBoost2 followed by RcisTarget to construct and filter scGRN per sub-sample. For each scGRN inference, a regression model is built for each gene that predicts its expression across cells based on the expression of predefined TFs. Every TF-Gene pair is assigned a weight quantifying the importance of each TF in predicting the expression profile of the gene. This produces a weighted list of adjacencies connecting a TF to genes (i.e. possible target genes). The weights are used to distinguish between strong and weak regulatory interactions. For module generation, GRNBoost2 employs multiple strategies to generate modules by converting the highest weights of each TF into TF-Gene regulatory links (43). We denote the resulting TF–gene networks as *e*_A_[*w_i,j_*]_*i*_, *e*_B_[*w_i,j_*]_*i*_ where *i* = 1,…,*K* for sample A and sample B, respectively and where W is the weight of the edge from *i* to *j*. The binary adjacency matrices are constructed by collapsing the sub-sampling scGRNs by taking the average weight across scGRNs (Figure 1C, D). Additionally during this process, an occurrence rate is associated with each edge representing the number of times this edge was detected in the results across all the sub-sampling scGRNs. These procedures are donated as follows:


$$\begin{equation*}{e_{i,j}} = \left\{ {\begin{array}{@{\quad}*{1}{c}@{\quad}} {1\,if\,i\,and\,j\,are\,connected}\\ {0\,otherwise} \end{array}} \right.\end{equation*}$$

The adjacency matrices are aligned in such a way that every edge (*i*,*j*) is associated with an occurrence rate.


$$\begin{equation*}{r_{i,j}}\left( \% \right) = \frac{{\sum {{e_{i,j}} = 1} }}{K} \times 100\end{equation*}$$

Users can set an occurrence threshold based on this rate to filter the low confidence edges as explained in [scGRNs alignments and denoising]. Following this filtering step all sample-based sub-networks are combined into a single weighted (i.e. mean of the weights across the sub-networks) TFs $ \times$ TGs adjacency matrix *E*_A_[*W*^A^_*i*,*j*_], *E*_B_[*W*^B^_*i*,*j*_].

#### Copula-based differential co-expression

In statistics, a common method to depict dependence between correlated random variables is by using Copulas (38). Copulas permit the estimation of the joint distributions from non-normal random variables, for instance, the expression profile of a gene across single cells. In principle, the joint distributions of any given gene expression profile can be modeled using Copulas.

The sample-based scGRNs are combined into one single adjacency matrix by taking the absolute value of weight difference *E*{*|W^A^_i,j_ – W^B^_i,j_|*}. *E* is a weighted TFs $ \times$ genes matrix where each non-zero entry represents a connected pair in the scGRN (*g*_i_*,g*_j_). We use Empirical Copulas (i.e. we selected a non-parametric Copula as the distributions of expression profiles are unknown) to model the differentiability by measuring the dependence between each pair of gene expression profiles.


(1) $$\begin{equation*}DiffSco_{i,j}^{s1,s2} = \left| {W\,{{\left( {T{F_i},\,T{F_j}} \right)}^{s1}} - W{{\left( {T{F_i} - T{F_j}} \right)}^{s2}}} \right|\end{equation*}$$

(2) $$\begin{equation*}DiffSco{\,_{{m_k}}} = \frac{{\mathop \sum \nolimits_{i,j \subset {m_k}} DiffSco_{i,j}^{s1,s2}}}{N}\end{equation*}$$

(3) $$\begin{eqnarray*} DiCoCopula\,{\left( {{g_i},{g_j}} \right)^{s1,s2}} &=& KS\left[ {C{{\left( {{x_{{g_i}}},{x_{{g_j}}}} \right)}^{s1}},C{{\left( {{x_{{g_i}}},{x_{{g_j}}}} \right)}^{s2}}} \right]\end{eqnarray*}$$

(4) $$\begin{eqnarray*} CopulaSimi\,{\left( {{g_i},{g_j}} \right)^{s1,s2}} &=& 1 - DiCoCopula\,{\left( {{g_i},{g_j}} \right)^{s1,s2}}\end{eqnarray*}$$

The following equations illustrate the differential co-expression procedure:

- In order to rank the modules, a differential score [*DiffSco*] is computed by taking the absolute values of the weights (i.e. already mentioned) [*W*] different in the two samples (*s*1, *s*2) [Equation (1)] (i.e. if the regulation relation does not exist in one of the samples the weight for that sample is set to zero). The module overall differential score is the sum of individual pairs differential scores divided by the number of pairs belonging to the given module [Equation (2)].
- The expression profiles (i.e. noted as *x*) of the gene pair are passed to the copula function [*C* in Equation (3)] to calculate the joint distribution in each sample. The Kolmogorov–Smirnov (K–S) test [KS in Equation (3)] is utilized to quantify the distance [DiCoCopula] between the two joint distributions. [Equation (3)]. Large [*DiCoCopula*] indicates increasing differential co-expression of the pair across the two samples. [*Copula*$Simi$] defines the similarity between a gene pair (i.e. TF and its TG) is formulated by [Equation (4)].

#### Derivation of statistical significance

Using a permutation test, we calculated an empirical one-sided *P*-value for each predicted pair using the following steps:

1. Randomizing the expression matrix while keeping the gene label fixed to get the randomized expression matrices *S*’^1^ and *S*’^2^ profiles (*g’*_i_*,g’*_j_).This is repeated *T* times.
2. Replacing the gene expression profiles in the Equation (3) with the randomized expressions *x_g’i_* and *x_g’j_* re-estimated the joint distribution from the randomized samples.
3. Driving distance based on Equation (4).

The *P*-value is calculated as follows:


$$\begin{equation*}P - value = \frac{{\mathop \sum \nolimits_i^T I\left( {CopulaSimi\left( {g_i^{\prime},g_j^{\prime}} \right) <\, CopulaSimi\left( {{g_i},{g_j}} \right)} \right)}}{T},i = 1,2, \ldots ,T\end{equation*}$$

where *I*(*i*) is the indicator function, it's equal to 1 if the condition in the parentheses is true, and 0 otherwise.

More detailed descriptions about the mentioned steps and methods above are given under the respective section below.

### DiNiro workflow

#### Single-cell data

Single-cell data can be imported to DiNiro as an AnnData object in H5AD file format (42). The input data must go through the typical single-cell data pre-processing steps, which include quality control, normalization, data correction, feature selection, and dimensionality reduction. The AnnData object allows the user to store the expression matrix and the preprocessing analysis outputs as metadata. When uploading the data into DiNiro, the user can access the stored metadata. We use the stored plots (e.g. tSNE, UMAP) as starting data, where cells are represented as points. The user can interact with the displayed data, color cells based on different conditions (cell type, conditions, sex, individual etc.), and select cell cluster (s) of interest to form two samples, which will be analyzed.

#### Subsampling

The data we are handling is often noisy and sparse despite the preprocessing and filtering. Furthermore, the single-cell data capture a temporal snapshot (44–46), where cells are in different states and phases, which leads to cell-to-cell heterogeneity and an abundance of intermediate states. In other words, the gene expression profiles vary even within the same cell type, which is a major obstacle in understanding the underlying regulatory dynamics within the data. To overcome this, we reasoned that randomly subsampling cells and learning multiple scGRNs and combining them into one large scGRN will permit us to eliminate the signals sourced from noise and outlier cells. Through the examination of multiple scGRNs, we can distinguish the conserved signals across the sample and the ones from noise. Subsampling is an important step to eliminate the noise and therefore accurate scGRN construction, which is crucial for the further steps.

#### scGRN construction

scGRNs define the ensemble of interactions among genes and TFs that govern their expression in single-cell expression data. Several computational approaches have been proposed to accomplish the task of uncovering co-expression patterns among regulators and their TGs using statistical or machine learning techniques (8,16–20). The inferred potential regulatory associations are displayed in GRNs. To date, scRNA-seq technologies enable parallel profiling of millions of cells. While the large data availability provides unprecedented opportunities to gain new insights by enhancing the quality and resolution of these network predictions, it turns the task of inferring scGRN into a computationally expensive task. This initiated the call for more efficient and scalable approaches that can carry out the task of deriving scGRN from large expression datasets.

Since we require inferring multiple scGRNs from multiple sub-samples (i.e. originated from the subsampling step), GRNBoost2 (11) was adopted to construct scGRN. GRNBoost2 is a regression-based GRN inference method and based on the same architecture as GENIE3 (21). GRNBoost2 is a faster alternative to GENIE3 due to its scalability, thus paving the way for network inference from large data sets. GRNBoost2 builds a tree-based regression model for each gene in the database with the aim of predicting the expression profile of the gene in the function of the expression profile of candidate regulatory genes (TFs). The regulators, whose expression profiles largely contribute to predicting the TG, are retained as candidate edges in the resulting GRN. Additionally, to exclude potential false prediction, we used RcisTarget (43,47) to perform *cis-*regulatory TF binding motifs enrichment analysis of each TF and its TGs (i.e. Module). RcisTarget validates for every edge in the GRN [TF, TG] if the regulatory binding motif for the TF is significantly enriched at the *cis*-regulatory region of the TG otherwise it is discarded as a potential regulator of the gene. The remaining edges construct the scGRN which is a direct graph (i.e. TF pointing to the gene orientation indicates regulation direction). This scGRN construction procedure (i.e. GRNBoost2 followed by RcisTarget) was adopted from pySCENIC workflow (43), where the authors seek to overcome general limitations of single-cell data by integrating *cis-*regulatory sequence analysis on top of co-expression analysis.

#### Alignment and denoising of scGRNs

A scGRN is computed for each sub-sample resulting from the subsampling step. Next, the sub-sample-based scGRNs are combined into one accurate scGRN. The alignment procedure associates an occurrence rate for every edge, which measures the frequency with which the edge occurs across all scGRNs. Considering that we are interested in capturing the biological insights that are homogeneous within each sample, edges with low occurrence rates mostly emerge from outlier or Inferior sets of cells, and those are to be filtered out. The remaining edges compose the final sample-based scGRN.

#### Copula-based model to identify differential co-expression

Copulas have functions that enable the construction of a joint distribution by combining the marginal distributions with a dependence structure. Copula was extensively explored for high-dimensional data (48–50). We make use of Copula to detect variation in co-expression patterns across two conditions based on gene expression profiles. Copula is used to model the dependency between gene pairs’ (i.e. TF and a TG) expression profiles in individual samples. This information serves as the basis for calculating the distance of the pair between samples. The distance between a gene pair represents the difference in the regulatory relationship between each regulator and its target across the two samples. In consideration of the fact that single-cell data is noisy and sparse, Copula performs well due to its scale invariance property. First, the expression vector (i.e. The gene expression profile across all cells belonging to the sample) of the regulator gene (TF) and of the TG are fed to an empirical copula model as marginals in order to compute the joint distribution in individual samples (i.e. capture the dependence between the TF and the TG in each sample). The measured K–S (51) distance between the two joint distributions represents the difference in co-expression of the pair across the two samples. In order to determine statistical significance for the computed distances, a permutation test is performed by randomizing the sample's expression matrices.

## RESULTS

### Assessment of model performance

In order to evaluate the performance and robustness of our methodology, we performed a three-level validation analysis: we first benchmark DiNiro against several state of the art GRN inference tools to illustrate the added value in terms of detection of differential gene regulatory interactions. Secondly, we evaluated the performance of DiNiro on simulated data with ‘known’ differential gene regulatory interactions. Lastly, DiNiro was applied to three real-world datasets of scRNA- or snRNA-seq data from cell culture (*in vitro*) to animal model (*in vivo*) to postmortem human tissue (*ex vivo*). Here, we retrieved (i) de novo differentially scGRNs driven by Nur77 and KDM2B in exhausted CD8^+^ T cells; (ii) revealed changes in gene networks regulated by E2F members of host cells in response to infection; (iii) and found an increase in scGRNs related to immunity and neuronal excitation of brain cell subtypes susceptible to autism.

#### Data acquisition and preprocessing

Four public scRNA-seq datasets have been used for the assessment of DiNiro (Table 1). A pre-processing pipeline was applied to each data independently. First, we filter genes that are expressed in less than (0.01 × number of cells). Next, we performed a highly variable gene detection analysis using the highly_variable_genes function in the python package scanpy (42), and the 2000 most variable genes were selected for network inference. Finally, the data were log_2_-normalized before applying the different network inference algorithms. All datasets are publicly available (52–54), including the simulated datasets (details are given on data availability statement).

**Table 1.** Details on datasets employed in this assessment

Data name	Sequencing technology	Tissue	Disease	Cell × genes	Publication/source
pbmc3k	10× Genomics	PBMCs from a healthy donor	Healthy	2638 × 1838	10× Genomics datasets
Szabo	10× Genomics	Respiratory airway	COVID-19	810 × 33 244	Szabo *et al.* (52)
Fawkner-Corbett	10× Genomics	Intestine	Normal	4211 × 33 178	Fawkner-Corbett *et al.* (53)
Madissoon	10× Genomics	Lung parenchyma	Normal	57019 × 24 817	Madissoon *et al.* (54)

#### Comparison with other single-cell GRN inference algorithms

The percentage of intersection (*pI*), defined below, has been employed here to assess the sensitivity to noise. Given two GRNs, *N*1 and *N*2 inferred respectively from scRNAseq clusters *C*1 and *C*2, with |*N*| being the number of regulatory edges in the network *N*, the *pI* is computed as:


$$\begin{equation*}pI\left( {N1,N2} \right) = \frac{{\left| {N1 \cap N2} \right|}}{{\min \left( {\left| {N1} \right|,\left| {N2} \right|} \right)}}\end{equation*}$$

Single-cell data is inherently noisy and known for its low signal-to-noise ratio. This noise is observed as heterogeneity between individual cells, which complicates downstream analyses of the single-cell RNA-seq data. To illustrate the ability of DiNiro to handle noise and reduce the amount of differential interactions inferred from cell-to-cell noise versus those inferred from true biological variability, we compared the intersection of interactions found when comparing two true single cell clusters with a random shuffle of the same two clusters. We generate the random clusters by mixing and shuffling the cells from the true clusters, and then split them randomly into two clusters that have the same size as the original clusters. The percentage of intersection (*pI*) of the results from comparing every two clusters (clusterA-true versus clusterB-true and clusterA-random versus clusterB-random) indicate the sensitivity of the method to noise. High *pI* between the true clusters and the randomly generated clusters suggest low sensitivity to noise. The following four single-cell network inference methods are considered in this evaluation: GENIE3, GRNBoost2, Context Likelihood of Relatedness (CLR) (55) and GeneNet (56). Noteworthy, GENIE3 and GRNBoost2 are the best performing GRN inference tools in the single-cell benchmark (30). The differential network is defined for these tools by simply taking the disjoint set of edges emerged from the results of the compared clusters. We ran all tools using the default parameters indicated by the authors, for DiNiro the default parameters are (number of subsamples = 4, sub-sampling size (%) = 70, occurrence threshold (%) = 70, significance cutoff = 0.05, more details on parameters selection and effect refer to supplementary. Notes: the effects of parameter settings on the analysis).

**Figure 2.**
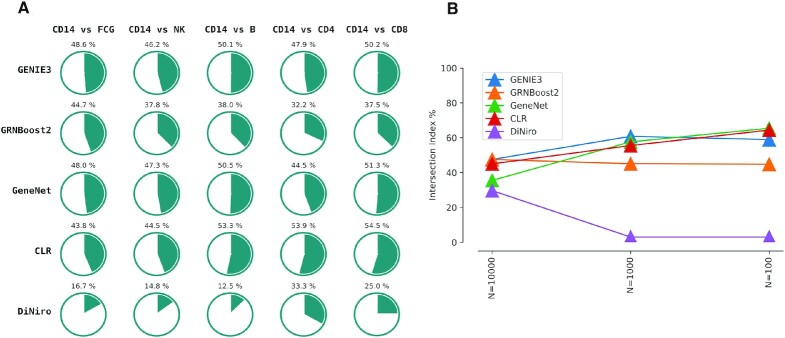
The performance benchmarking results reported for various algorithms. (**A**) benchmarking using varying cell type cluster comparisons from the pbmc3k dataset. (**B**) benchmarking using datasets with different cell clusters sizes.

The cluster CD14^+^ monocytes in the pbmc3k dataset was compared to all other cell types within the PBMC data (Figure 2A). The random data was generated for every comparison as described above, and the same data was used to benchmark all the tools. DiNiro outperformed them all by a lower *pI* across all comparisons (lowest 16.7% and highest 25%) followed by GRNBoost2 (lowest 37.5% and highest 44.7%) (Figure 2A). GENIE3, GeneNet, and CLR all showed a pI around 50% in all cases (lowest 44.5% and highest 54.5%). We further evaluated the scalability of DiNiro by using different cell cluster sizes *N* (*N* = 100, *N* = 1000, *N* = 10 000) (Figure 2B). The true and random clusters were generated as described above. For each case, the compared clusters are derived from a single cell dataset as follows: *N* = 100 the clusters [‘neutrophil’,‘mature NK T cell’] from Szabo dataset, *N* = 1000 the clusters [‘interstitial cell of Cajal’,‘smooth muscle cell of small intestine’] from Fawkner-Corbett dataset, and *N* = 10 000 the clusters [‘pneumocyte’,‘fibroblast’] from Madissoon dataset. Overall, DiNiro had a lower *pI* compared to the other methods regardless of the size of the compared clusters, and emerged again as the best performing method in all cases (*N* = 100, *N* = 1000, *N* = 10 000) (Figure 2B). For large cell cluster size *N* = 10 000 an increase in *pI* is observed for DiNiro, which can be justified by the large noise signals that are introduced when randomizing cells at such scale.

#### Simulated data

To further evaluate our methodology we designed study cases using synthetic datasets with known regulatory interactions. The simulation scheme begins by generating count data from marginal zero-inflated negative binomial distributions via the NORmal To Anything (NORTA) algorithm (57). The NORTA algorithm generates a standard normal random vector with a given dependency structure, and then transforms it into a random vector with specified marginal distributions, thus allowing to specifically induce certain TF–TG correlations. Counts were generated with the rnorta function from the R package SimCorMultRes (58), and the ZIM package (59) was used to estimate the parameters of the zero-inflated negative binomial distributions.

**Figure 3.**
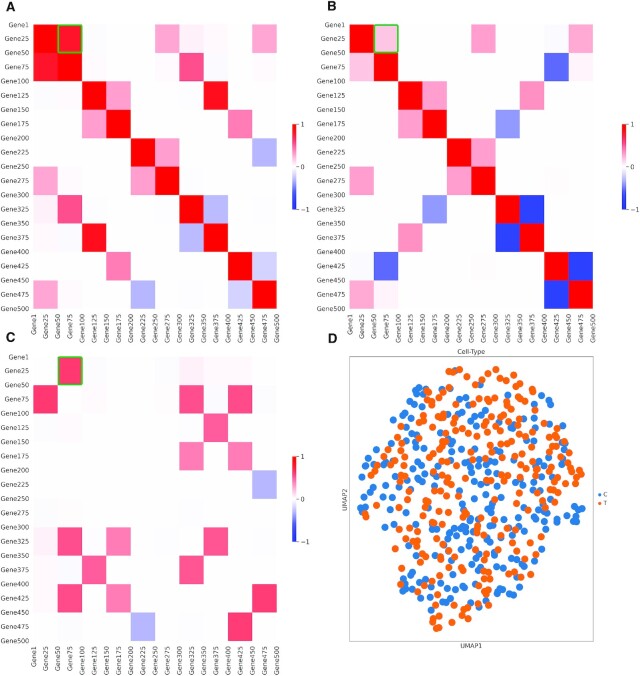
Schematic illustration of simulated data containing 500 cells and 500 genes. (**A**, **B**) Generated correlation structure of 500 genes split into groups of 50 for two clusters of each 250 cells. (**C**) The differences in correlation for each gene group between the two cell clusters. The green box in each heatmap highlights the ground truth gene group. (**D**) A UMAP plot of the final generated single cell data 500 cells x 500 genes.

We simulated three datasets with different sizes, but each data consists of the same number of cells *M* and number of genes *N* (*M* = *N* = 500, 1000 and 2000). In all three datasets, we supervised the correlation structures by sorting the genes into two main groups of size *N* (e.g. in the case of *M* = *N* = 500). Group-1 consists of gene 1–500, Group-2 consists of gene 1–500 (Figure 3A, B). Moreover, genes within each group were divided into groups of 50 genes in such a way that genes within the same group are highly correlated, and their intra correlation is structured (Figure 3A, B) (same structure used for other datasets). In all datasets, we used the gene group 51–100 as our true differential group, hence the difference in correlation between the genes in this group is augmented between the two main groups (Group-1, Group-2; Figure 3C). In each dataset, cells were divided into two equal clusters (Cluster-1, Cluster-2) of size *N*/2, and cells from each cluster were assigned the genes groups (Group-1, Group-2) as expression profiles (Cluster-1 & Group-1, Cluster-2 & Group-2). Once the simulated single-cell count data was generated, we changed gene names to known gene IDs, and the gene group 51–100 consisted of the transcription factor JUNB and 49 of its known target genes. The cells from Cluster-1 are annotated as control (C) and Cluster-2 are annotated as treatment (T) to stimulate the treatment group, for instance the UMAP plot of the data in case of *N* = *M* = 500 (Figure 3D).

The simulated datasets were analyzed using DiNiro, to evaluate its capacity of capturing the induced differential regulatory interaction between control and treatment cell clusters in each dataset. Using different data sizes ensures assessing the method's scalability. The default run parameters were used to run DiNiro (number of subsamples = 4, sub-sampling size (%) = 70, occurrence threshold (%) = 70), the significance cut-off of *P*-values were toggled between 0.01 and 0.05 to illustrate its effect on the results. Amongst the error matrix statistics, True positive rate (TPR) and the false discovery rate (FDR) are the most informative rates for evaluating the number of edges that were predicted as significantly different. The measure of TPR, FDR, and the number of edges that were predicted as significantly different between the cell clusters (control and treatment) for each simulated data are displayed in (Table 2). Overall, our method performed well (average TPR = 0.798) in capturing most of the induced interactions. TPR seems to slightly increase proportionally with the dataset size (0.75 in case of *N* = *M* = 500 to 0.937 in case of *N* = *M* = 2000). This is due to the availability of more cells for the subsampling process used in DiNiro which in turn enhance the performance. In general, DiNiro tends to be a bit more conservative and detects fewer significant edges when lowering the p-value threshold.

**Table 2.** TPR and FDR according to different simulated data sizes are reported for varying values of the *P*-value cut-off

Simulated data (*M*x*N*)	*P*-value cut-off	TPR	FDR	Diff. edges
SimData-1 (500 × 500)	0.01	0.625	0.268	41
	0.05	0.75	0.25	48
SimData-1 (1000 × 1000)	0.01	0.791	0.344	58
	0.05	0.833	0.322	59
SimData-1 (2000 × 2000)	0.01	0.85	0.25	55
	0.05	0.937	0.196	56

**Figure 4.**
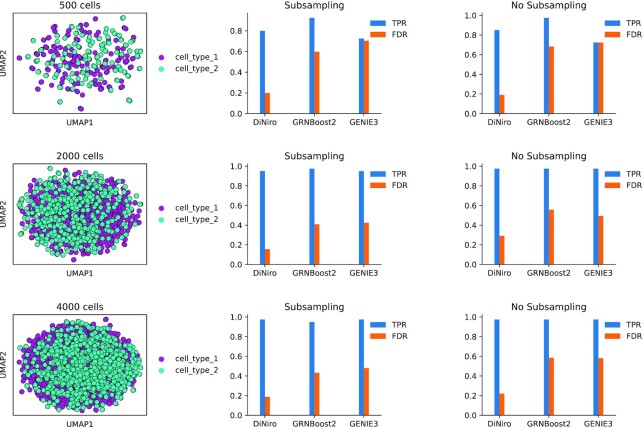
Summary of benchmarking results for three simulated single-cell datasets with and without subsampling. Each row displays the simulated data UMAP, followed by a bar plot showing the performance of the compared tools measured using TPR and FDR rates in both cases with and without subsampling.

To further demonstrate DiNiro's power in capturing differential GRN between two cell types and lowering false positive predictions, we compared its performance on simulated data to GENIE3 and GRNBoost2 (using the implementation of pySCENIC). Since these tools are designed for global GRN inference, we define the differential network as the difference between the GRN networks inferred from the two cell types. We used SERGIO (60), a tool capable of simulating realistic single-cell transcriptomics datasets using a user-specified gene regulatory network, to simulate three single-cell datasets (Figure 4) each containing two cell types (GRN). SERGIO-simulated datasets are intended to test a range of single-cell analysis tools, including GRN inference approaches.

We simulate single-cell data in such a way that a GRN comprising two modules is activated in one cell type while suppressed in the other. This GRN serves as a ground truth for the differential GRN. The two modules constituting the GRN have different sizes 10 and 30 to simulate the real biological data. SERGIO was configured with the following parameters: noise params = 1, decays = 0.8, sampling state = 15, noise type = ‘dpd’. To generate three datasets of varying sizes, we utilized number genes = 250 and three distinct cell counts of 500, 2000 and 4000 in all simulations.

Additionally, to illustrate the effect of the subsampling step on filtering false positives and predictions originating from the outlier in the data, we benchmarked the tools with and without this step. In the case of subsampling these parameters were used for all tools four subsamples with 70% subsampling size and 100% occurrence threshold and for DiNiro the *P*-value cut-off was set to 0.01. TPR and FDR rates were used to evaluate the tools, all the edges in the ground truth are sought and considered as true positives any extra edges predicted are considered false positives. The results of this benchmarking are shown in (Figure 4), where each row shows the UMAP plot of the simulated data, as well as the TPR and FDR bar plots of each tool with and without subsampling.

Overall, all methods yielded adequate TPR rates, which increase in direct proportion to data size, as additional data amplifies the signal. DiNiro generally outperforms in terms of FDR rates (FDR < 0.2), independent of data size. When no subsampling is used, the performance declines (higher FDR rates), suggesting that this step is effective in filtering out predictions arising from noise and outliers within the data and therefore minimizing false positives. The benchmarking results show that employing DiNiro helps to eliminate false positives and obtain more accurate differential scGRN.

#### Case study

To further examine our proposed methodology, we analyzed three real-world single-cell data from: (i) molecular mechanism(s) of T cell fate after chronic viral infection, (ii) the cellular dynamics in the interactome of host cells infected with SARS-CoV-2 and (iii) pathological networks in subtypes of brain cells susceptible for changes in autism (Supplementary Materials S1).

##### Network modeling of long-term antiviral CD8^+^T cells: the key gene regulatory differences between cell fate of stemness vs terminal exhaustion

**Figure 5.**
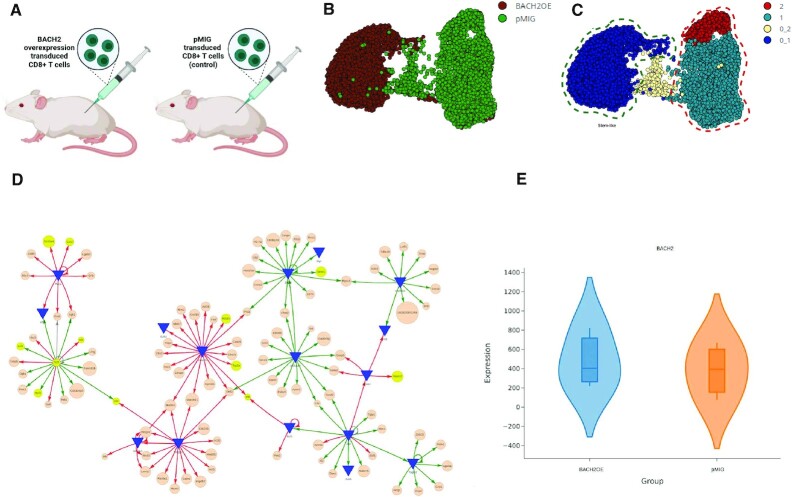
BACH2 overexpression promotes stem-like CD8^+^ cell clusters with different molecular interactions. (**A**) Experimental setup (Yao *et al.* 2021): CD8^+^ T cells with BACH2 overexpression (BACH2 OE) or CD8^+^ T cells with pMIG empty-vector control/ (pMIG) both transferred to mice infected with LCMV clones. (**B**) UMAP of snRNA-seq data collected from CD8^+^ T cells of pMIG and BACH2 OE mice on day 7 post-infection. (**C**) Unsupervised identification of cell subsets and selection of stem-like cell cluster (0_1) and terminal exhausted cell clusters (1,2) for network modeling in DiNiro. (**D**) *De novo* reconstruction of transcriptional gene regulatory network modules with DiNiro using significant cut off 0.1 for five subsamples with 70% subsampling size and 90% occurrence threshold. Green interaction lines are significant for stem-like cell cluster (0_1), red interaction lines for terminal exhausted cell clusters (1,2). Discovered and discussed genes from the original study (Yao *et al.* 2021) are highlighted in yellow. Interactive network visualization S1 Table A. (**E**) Violin plot of BACH2 expression in the compared scRNA-seq data samples.

**Figure 6.**
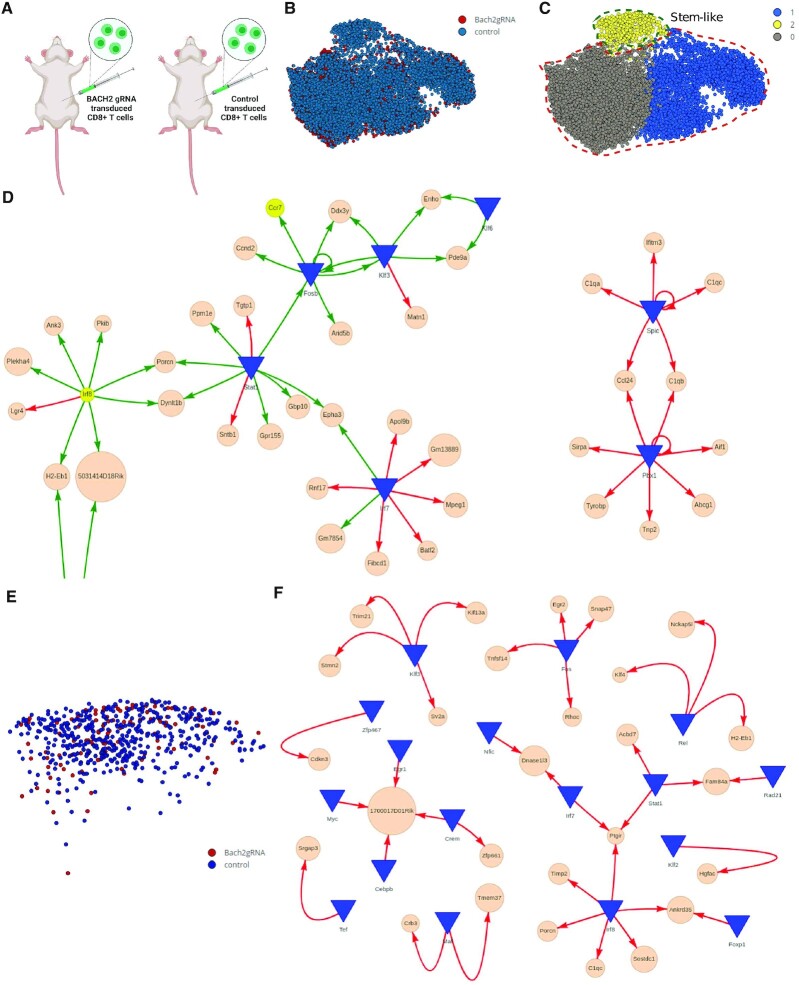
Insufficient stem-like response in virus-specific CD8^+^ cells lacking BACH2 gene. (**A**) Experimental setup (Yao *et al.* 2021): CD8^+^ T cells transduced with control or Bach2 gRNA constructs were adoptively transferred into C57BL/6 mice that were subsequently infected with LCMV clones. (**B**) UMAP of snRNA-seq data collected from CD8^+^ T cells from Bach2gRNA and control on day 7 post-infection. (**C**) Unsupervised identification of cell subsets, and selection of stem-like cell cluster (2) and terminal exhausted cell clusters (0,1) for network modeling in DiNiro. (**D**) A *de novo* reconstruction of transcriptional gene regulatory network modules was produced with DiNiro using significant cut off 0.1 for 5 subsamples with 70% subsampling size and 90% occurrence threshold. Green interaction lines are significant for stem-like cell cluster (2), red interaction lines for terminal exhausted cell clusters (0,1). Discovered and discussed genes from the original study (Yao *et al.* 2021) are highlighted in yellow. (**E**) Visualization of the cells from Bach2gRNA (brown) and controls (black) in the stem-like cell cluster. (**F**) *De novo* reconstruction of transcriptional gene regulatory network modules with DiNiro comparing controls (red interaction lines) and Bach2gRNA (green interaction lines). Network parameters are the same in (D). Interactive network visualization S1 Tables B and C.

To demonstrate the power of DiNiro, we used the scRNA-seq data from Yao *et al.* (61). They focused on two CD8^+^ T cell populations: (i) the terminally exhausted CD8^+^ T cells, a homogeneous population defined by Ly108^low^TIM-3^hi^ and characterized by a specific irreversible epigenetic imprint (62,63) and (ii) the stem-like CD8^+^ T cells defined by Ly108^hi^TIM-3^low^ with a distinct lineage from all other viral-specific CD8^+^ subtypes, and suggestive to be a crucial player against chronic infection and cancer (64–66). They showed that the gene encoding BACH2 enforces stem-like cell fate of CD8^+^ T cells in mice during chronic viral infection. They established this discovery using snRNA-seq on splenic CD8^+^ T cells with either BACH2 overexpression (Figure 5A) or BACH2 deletion (Figure 6A) compared to wildtype (WT) CD8^+^ T cells. Both were transferred to mice infected with lymphocytic choriomeningitis mammarenavirus (LCMV), a widely used model system for chronic infections and cancer studies.

To sort out the effect of BACH2 overexpression on the transcriptional program of virus-specific CD8^+^ T cells, they performed unsupervised clustering dividing the cell groups in three (four): 0 (0_1, 0_2), 1 and 2 (Figure 5C). They found upregulation of stem-cell markers in cluster 0 representing almost all BACH2 overexpressing cells, while makers of T cell dysfunction and terminal exhaustion were higher expressed in clusters 1 and 2 (Figure 5C). To further understand the involved molecular networks, and the effect of interactions on a global scale with overexpression of BACH2, we constructed the AnnData object from their single cell gene expression data (11 589 cells, 2000 genes), and uploaded the data to DiNiro (Figure 5C). The authors of this study used the top 2000 variable genes for the analysis. We followed the same data pre-processing to be able to compare our results with the original results of the paper. The significant cutoff was 0.1, and the run parameters were set for five subsamples with 70% subsampling size and 90% occurrence threshold (S1 Table A). Differential submodules of gene interactions between the two cell clusters were present in the generated DiNiro network (Figure 5D, interactive network visualization S1 Table A). Genes related to cell cycle and DNA damage (TOP2A, H2AFX), marker of terminally exhausted CD8^+^ T cells (HAVCR2) and chemokine receptors (CCR2) were associated with networks in clusters 1 and 2, as in the original study (61) (Figure 5D, Table 3). In addition, the DiNiro networks also uncovered several molecular interactions in mouse CD8^+^ T cells: (i) identifying the regulatory factor of HAVCR2, the NR4A1 gene (also called Nur77), which is a known critical mediator of T cell dysfunction and exhaustion in chronic inflammation and cancer (67,68). (ii) An exhausted cluster of genes related to DNA damage and cell cycle regulated by KDM2B encoding lysine demethylase 2B. KDM2B target genes included among others apoptotic-related genes (FASL, TMPO) and a cytokine (CXCL16) (Figure 5D). KDM2B is known to enhance terminally Th17 cell formation (69) and is involved in maintenance of T-cell acute lymphoblastic leukemia (70), which all could support the role of a phenotypic dysfunctional exhausted T cell type in the clusters without BACH2. (iii) TCF7 was a major hub in a submodule with genes overrepresented in the stem-like cell cluster (cluster 0) (Figure 5D). TCF7 is mentioned in the original paper as a significant signature of this stem-like cell type (61). Tcf7 is connected to additionally 16 genes in the DiNiro network (Figure 5D), where five are also found in the original paper (61). Two of them (IKZF2, VIM) were also expressed in this cell cluster, while another (Lef1), they detected in their bulk RNA-seq data from BACH2 overexpressed cells.

**Table 3.** Genes and their known biological function detected in *de novo* reconstruction of transcriptional gene regulatory network modules with DiNiro in the stem-like cell cluster (0_1, 0_2) and in the terminal exhausted cell clusters (1,2)

Cluster 1 and 2	Biological function	Genes
Findings by the original paper and DiNiro	DNA damage	Top2a,H2afx
	Terminally exhausted CD8 + T cells	Havcr2
	Chemokine receptor	Ccr2
Additional findings by DiNiro	Critical mediator of T cell dysfunction and exhaustion	Nr4a1
	Regulator of terminally Th17 cell formation	Kdm2b
	Apoptotic-related genes	Fasl,Tmpo, Bcl2
	Cytokine	Cxcl16
Cluster 0_1 and 0_2	Biological function	Genes
Findings by the original paper and DiNiro	signature of this stem-like cell type	Tcf7,Ikzf2,Vim
Additional findings by DiNiro and bulk RNA data	signature of this stem-like cell type	Lef1
Additional findings by DiNiro	Embryonic development	Lfng
	Cell cycle	Ccna2,Cenpe, Ccnd2
	Immune-related genes	Cd74,Socs1, Peli1

Noteworthy, the mean BACH2 is not efficiently overexpressed across all the CD8 + T cells of the BACH2OE samples (Figure 5E), which may explain overlapping edges from both populations in the connections of the GRN of the stem-like signature, TCF7 (Figure 4D). However, the experimental setup seems sufficient to generate two distinct T cell populations with a stem-like signature versus terminally exhausted signature (61).

To sum up this part, by using DiNiro, we have been able to discover the regulatory networks of NUR77 and KDM2B as potential key drivers behind the genetic profile of terminally exhausted CD8^+^ T cells for the first time. These predictions can foster novel hypotheses and experimental designs. This emphasizes how DiNiro can be applied to acquire more than just cell clusters and gene lists, but also molecular connective information with de novo reconstruction of transcriptional gene regulatory network modules.

Next, Yao *et al.* used a retroviral CRISP-cas9 system to delete Bach2 in activated CD8^+^ T cells in mice to determine BACH2 deficiency on the transcriptome during chronic viral infection (Figure 6A). Their single cell gene expression data (9621 cells, 1999 genes) was processed and uploaded in DiNiro (Figure 6B, C). With unsupervised clustering three distinct cell clusters were presented, where cluster 2 was identified as the stem-like CD8^+^ T cell type (61) (Figure 6C). In the network, we identified subnetworks of TFs regulating both genes that dominate in the defined stem-like cell cluster or in the defined exhausted cluster from the original paper (Figure 6D, S1 Table B). A major hub, IRF8 was regulating six additional genes present in cluster 2, the stem-like cell cluster. IRF8 is a TF important for controlling immune responses in T cells (71). Based on ATAC-seq and ChiP-seq experiments, Yao *et al.* also detected this transcriptional motif highly active in the stem-like cell type (61).

We focused on the stem-like cell cluster, even though BACH2 deficiency did not overtly separate into a distinct cluster (Figure 6B). However, Yao *et al.* (61) found differentially expressed genes between BACH2 deficiency and control in the stem-like cell cluster (61). Thus, we next focused on the stem-like cell cluster and compared BACH2-deficient and control cells in DiNiro using the same parameters as previously (Figure 6E). The resulting networks only contained modules present in the control cells. Among those were early growth immune response proteins (MAF, FOS) supporting Yao's findings for stem-like cells (Figure 6F, S1 Table C). The lack of differentially signaling networks in the stem-like cells without BACH2 compared to stem-like cells with BACH2 suggest specific insufficient signaling networks in the absence of BACH2. This highlights the power of DiNiro in detecting the differences in network connectivity between two similar cell types by focusing on differential GRNs while neglecting the homogeneous/similar ones.

##### Network modeling of SARS-CoV-2 infected cells

**Figure 7.**
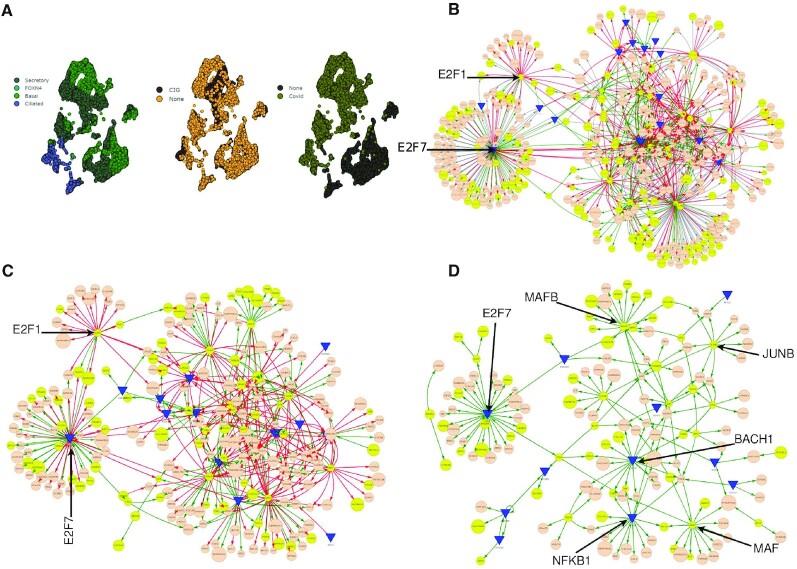
Network-based SARS-CoV-2 infection profile of cells from air-liquid interface (ALI) cultures derived from airway basal stem cells (ABSCs). (**A**) UMAP of snRNA-seq data colored by: cell type (secretory, FOXN4+, basal, ciliated cells); exposure to cigarette smoke(CIG); or infected with SARS-CoV-2 (CoV-2). (**B**) DiNiro transcriptional gene regulatory network modules using significant cut off 10^−28^ for four subsamples with 50% subsampling size and 100% occurrence threshold. Green interaction lines are significant for SARS-CoV-2 infected cells, red interaction lines for non-infected cells, and gray lines for shared interactions. Detected significant genes from the original study (Purkayastha *et al.*, 2020) are highlighted in yellow. Arrows show two major hubs (E2F7, E2F1) dominating in either infected or non-infected cells. (**C**) Same network as in (B) without shared interaction. (**D**) UMAP of (B) only with interactions of SARS-CoV-2 infected cells. Arrows highlight the E2F7 a hub in the major submodule, and additional hubs that control inflammatory responses. The networks shown in (B–D) can be explored in full size as DiNiro output. Interactive network visualization S1 Table H.

In the next case example, we wanted to use DiNiro for discovery of how host cells’ interactome changes in response to infection with SARS-CoV-2. Therefore, we used the dataset from Purkayastha *et al.* (72) to search for dynamic alterations in gene regulatory networks of air–liquid interface (ALI) cultures derived from Airway Basal Stem Cells (ABSCs) of five healthy lung transplant donors (72). In the clustered data, all major cell types (ciliated cells, basal cells, secretory cells, FOXN4^+^ cells) were present with and without exposure to cigarette smoke (CIG) (Figure 7A). Nevertheless, the most distinct separation was cells with or without SARS-CoV-2 infection (Figure 7A).

We generated the gene-regulatory networks of SARS-CoV-2 infected cells vs non-infected cells (19361 cells, 2000 genes, *p* = 10**^−^**^28^ subsamples = 4, subsampling size = 50%, occurrence threshold = 100%, S1 Table H). Regardless of conservative significant threshold and 100% occurrence threshold, the networks were huge including several genes also detected in the original paper (yellow nodes) (Figure 7B). It comprised three overall submodules, which were consistent when removing all shared interactions (Figure 7C). The biggest submodule contained several hubs with a mix of interactions between genes unique for non-infected or infected cells, highlighting the impact of SARS-CoV-2 on the overall molecular connections and cellular pathways. Several nodes were genes coding for proinflammatory cytokines, chemokines and metalloprotease and membrane proteins involved in activating immune responses (TNF, IRF7, CCL4, CLL5, IL6, CD14, ILR7, MMP2, IL11, IL24, CXCL13, CD53, CD70, CXCL11, VCAN, CXCL14, HLA-DRB5, TNFSF13B, CD7, CHI3L2, VCAM1), supporting how the host immune dynamics is highly activated in response to SARS-CoV-2 (Figure 7C).

The two other networks were more specific for either infected or non-infected cells regulated each by one TF, which are both members of the E2F family (Figure 6C). Together, E2F members control the expression of many genes that are needed for cell proliferation, but they do also have additional properties including regulating cell differentiation and cell survival (73). E2F1, dominating in the non-infected cells, is a potent promoter of apoptosis, but also involved in regulating cell metabolism and a player of DNA repair (74–76). In the other submodule for the infected cells, E2F7 was the major hub (Figure 7C, D). E2F7 has a critical repressive function of the E2F1-apoptotic-dependent network and constrains against excessive E2F1 activation (77,78). E2F7 is also known to modulate DNA repair and promote cell proliferation and differentiation in a variety of cancer types (79–82). Therefore, one could speculate that during SARS-CoV-2 infection, the virus may interfere with the E2Fs genetic programming towards an inhibition of cell death and promotion of cellular proliferation through E2F7 to ensure viral replication and productive infection.

Additionally, in the gene-regulatory networks unique for the infected cells, several hubs were TFs that play critical roles in immune responses (NFKB1 (82), MAFB (83), MAF (84), BACH1 (85), JUNB (86)) (Figure 6D), supporting a highly immunological cellular host response, when infected with SARS-CoV-2.

In all, these networks may help to define molecular aspects of pathogenic phenotypes in SARS-CoV-2 infected cells, and identify pathways that can be used to explore first of all the host responses that support (proviral factors) or restrict (antiviral factors) infection, but also potential networks manipulated by the virus. Note, that conclusions drawn with the help of DiNiro need experimental validation and can guide future studies by proposing novel hypotheses on molecular disease mechanisms.

DiNiro was also used to model networks of specific brain cell types predictive of clinical autism severity using single nuclei RNA-seq (snRNA-seq) data. This study details and findings are detailed in (Supplementary S1 and Supplementary Figures S1 and S2).

## DISCUSSION

In line with the rapid development of single-cell sequencing technologies, several methods for gene-level downstream analysis of single cells have been developed over the last few years. Yet, most of these methods were dedicated to differential gene expression and co-expression analysis, while little attention was paid to differential co-expression. The latter holds enormous potential for understanding the molecular mechanisms governing cells, which facilitates the study of biological processes, diseases, and phenotypic variations by finding modules of genes whose co-expression patterns vary across conditions.

To address this increasing need in research, we present DiNiro, a framework for comparing two single-cell samples/groups/conditions at the gene level, which is based on gene regulatory networks and copulas. Starting from two single-cell samples DiNiro performs a complete differential co-expression analysis process and outputs the differentially regulated gene across the samples in the form of modules. Furthermore, the detected modules undergo a gene set enrichment analysis to ease retrieving the functional profile of the modules in order to better understand the underlying biological processes. DiNiro serves as an intuitive interactive platform for reconstructing and identifying transcriptional gene regulatory network modules that differentiate cell clusters. By comparing single-cell samples of interest, researchers can detect the gene that vanishes or switches regulation across samples or clusters organized as modules. This facilitates exploring the results and focuses on modules that contain more relevant biological information. In all, this will assist in understanding the gene expression mechanism inside cells, and promote the research of disease pathology at the level of a single cell.

In addition to evaluating the robustness and scalability proposed methodology on a simulated datasets, we applied DiNiro on three studies and showed how the tool is a valuable addition to the interpretation of scRNA- or snRNA-seq data. Here, we discovered multiple gene regulatory networks, and it allowed us to gain deeper insights into (i) molecular mechanism(s) of T cell fate after chronic viral infection, (ii) the cellular dynamics in the interactome of host cells infected with SARS-CoV-2 and (iii) pathological networks in subtypes of brain cells susceptible for changes in autism (Supplementary S1).

Our results demonstrate that the investigation of complex scRNA data through systematical mapping of differentially expressed gene regulatory modules between different biological conditions can therefore aid to both explore novel relevant biological connections and to discover potential target pathways for development of diagnostic, therapeutic and preventive measures in the fight against complex disease.

Single-cell RNA-seq regulatory networks have more false-positive edges than bulk RNA-seq regulatory networks (7). This is mainly due to increased technical noise, biological variations (e.g. stochastic transcription), and the sparse nature of scRNA-seq data, which poses significant challenges for GRN inference analysis. DiNiro employs various strategies to reduce false positives, including inferring multiple GRNs and filtering GRNs for putative direct-binding targets based on TF binding motif enrichment. When compared to current technologies, this considerably reduces false positive rates (Figure 4). Users should be aware, however, that inferred modules may still contain a few false positive edges.

DiNiro performance is heavily based on the GRN inference accuracy, and it outperforms the current best performing GRN inference tools. However, in general this task is still challenging due to the high signal-to-noise ratio in single-cell data and unsynchronized transcriptional dynamics across cells, which may explain an average FDR = 0.271 for significant predicted edges. Upon the availability of more robust methods for GRN inference from single-cell data, those will be integrated into the future versions of DiNiro to boost the performance. In the future, extensions of DiNiro could cover additional aspects of single-cell OMICs, for instance, multi-species data analysis.

## DATA AVAILABILITY

All the three used datasets are openly available in a public repository. The data for the long-term antiviral CD8+ T case study were downloaded from the Gene Expression Omnibus (GEO accession no. GSE152379). The data for the clinical autism severity case study were downloaded from the Sequence Read Archive (SRA accession no. PRJNA434002). The data for the SARS-CoV-2-infected cells case study were downloaded from Gene Expression Omnibus (GEO accession no. GSE161089).

DiNiro is freely available as an online tool at https://exbio.wzw.tum.de/diniro/ and can be downloaded as a standalone program from GitLab (https://gitlab.com/mhanedd/diniro). The generated simulated data and the script used to generate it (Jupyter Notebook) is available also within the diniro gitlab repository as h5ad files under the folder [simulated data]. Full DiNiro output for the discussed case study are given in the supplementary materials. The datasets and the tool can be used to reproduce all experiments and results in this paper using the same given parameters.

## Supplementary Material

lqad018_Supplemental_File
