# Supplementary material for: De-novo reconstruction and identification of transcriptional gene regulatory network modules differentiating single-cell clusters
Source: NAR Genom Bioinform. 2023 Mar 3;5(1):lqad018. doi: 10.1093/nargab/lqad018 (PMC9985332; doi:10.1093/nargab/lqad018)
Supplement: lqad018_Supplemental_File [file lqad018_supplemental_file.docx]

# **Contents**

**1. Case Study: Network modeling of specific brain cell types predictive for clinical autism severity**

**2. Supplementary Tables**

**3. Supplementary Notes**

# **1. Network modeling of specific brain cell types predictive for clinical autism severity**

To demonstrate how DiNiro also can be used on single nuclei RNA-seq (snRNA-seq) from sparse samples as human postmortem brain samples, we used data from 15 autism spectrum disorder (ASD) and 16 controls [(1)](https://paperpile.com/c/RLgKWZ/934QJ). In this study, Velmeshev et al. found 17 cell clusters, where differentially expressed genes (DEGs) were related to dysregulated development and synaptic signaling especially from upper-layer cortical circuits (L2/L3 neurons), as well as changes in cellular state of glia. The most predictive of clinical severity were not among the top ASD DEGs, but among changes in L2/3 neurons and microglia. Therefore with DiNiro, we wanted to further investigate the differences in the molecular network within those cell types.

### **Microglia in ASD patients: different aging- and immunophenotype**

We extracted the microglia cluster (3331 cells, 2043 genes), and created submodules from ASD patients vs controls in DiNiro (parameters: p=0.2, subsamples=4, subsampling size =70%, occurrence threshold = 90%) (Fig S1A S1 Table D). Here, we focused on four subnetworks that included DEGs also reported in the original paper (Fig S1B) (<https://autism.cells.ucsc.edu>) [(1)](https://paperpile.com/c/RLgKWZ/934QJ). The major subnetwork unique for ASD brains is controlled by SREBF2 (Fig S1B). SREBF2 is a TF that induces genes involved in cholesterol and fatty acids biosynthesis. SREBF2 and other TFs in the biosynthesis are known to be activated by antipsychotic drugs [(2, 3)](https://paperpile.com/c/RLgKWZ/dtBDT+JYhRQ). If the patients have been treated (data not shown in Velmeshev et al., Science, 2019), one could speculate whether this molecular network reflects the drug mechanism of action rather than the disease mechanism. The other subnetwork only present in nuclei from ASD brains was regulated by CREB3L2, which is also reported to co-express in different cells of the CNS [(4)](https://paperpile.com/c/RLgKWZ/AbSqq). It is connected to GPX1 and EIF1 both found downregulated in reactive neuroprotective astrocytes[(5)](https://paperpile.com/c/RLgKWZ/c7Byg) and both are known to protect from cell aging [(6)](https://paperpile.com/c/RLgKWZ/UZhFP).

Additionally, a subnetwork contains the IRF8, a TF also present in the CD8^+^ T cells (Fig. 5). IRF8 regulates critical roles in maturation and differentiation in a broad range of immune cells [(7)](https://paperpile.com/c/RLgKWZ/ScxNQ). Microglia are the resident myeloid immune cells of the brain, and IRF8 plays a major role in transforming microglia to their reactive phenotype [(8)](https://paperpile.com/c/RLgKWZ/C7zIL). In the nuclei from ASD patients it regulates LST1, a microglial gene involved in phagocytosis and has been reported in other neurological diseases such as Alzheimer and multiple sclerosis [(9–11)](https://paperpile.com/c/RLgKWZ/TPr1E+jjpaH+94bj2). In the non-diseased nuclei, IRF8 is connected to the ADCY7, which is associated with microglia aging [(12)](https://paperpile.com/c/RLgKWZ/ngA8x).

Including DiNiro to the snRNA microglia cluster, we found that microglial networks in ASD patients may be transformed towards a more immune activated phenotype with increased lifespan. This is in line with findings of excessive microglia activation from brain imaging of ASD patients [(13)](https://paperpile.com/c/RLgKWZ/yiGAN), and that a specific immune phenotype (M2-activation state) of microglia is altered in ASD [(14)](https://paperpile.com/c/RLgKWZ/nf74T).

**Fig S1. Microglia networks in autism spectrum disorder (ASD) vs non-diseased human brain tissue.** (A) Extracted microglia cluster from snRNA-seq data of 15 autism spectrum disorder (ASD) (blue nuclei) and 16 control postmortem brain tissue (purple nuclei). (B) Selected DiNiro transcriptional gene regulatory network modules using significant cut off 0.2 for 4 subsamples with 70% subsampling size and 90% occurrence threshold. Green interaction lines are significant for ASD microglia, red interaction lines for non-diseased microglia. Detected significant microglia genes from the original study (Velmeshev et al. 2019) are highlighted in yellow. (C)Extracted microglia cluster as in (A). Nuclei from females colored in pink and nuclei from male in purple. (D) Selected DiNiro network modules using significant cut off 0.1 for 4 subsamples with 70% subsampling size and 90% occurrence threshold. Green interaction lines are significant for female microglia, red interaction lines for male microglia. Shared interactions have been removed (gray lines). Interactive network visualization S1 Tables D and E.

### **Sex-specific cell differences**

Since, there is a higher incidence of ASD in men (4:1)[(15)](https://paperpile.com/c/RLgKWZ/UCfZZ), and recent studies have shown that microglia are physiological and transcriptional different between males and females, we wanted to seek out, if DiNiro could detect differences in male and female microglia [(16, 17)](https://paperpile.com/c/RLgKWZ/Oq7da+XpDot). Therefore, we compared female microglia with male microglia (parameters: p=0.1, subsamples=4, subsampling size =70%, occurrence threshold = 90%) (Fig S1C). We found 20 modules different between males and females (Fig S1D S1 Table E). This included microglia-immune related modules (HLA-C, AIF1 NFKB1, TNFRSF1B, MRC2) and oxidative stress responses (NFE2L2, HIF1A, CUBA, NRF1) supporting the sex-related differences in key cellular functions in microglia[(16, 17)](https://paperpile.com/c/RLgKWZ/Oq7da+XpDot).

Next, we examined L2/3 neurons, the other cell type that correlated with disease severity (Fig S2A). Velmeshev et al. found this cell type to have the largest number of DEGs [(1)](https://paperpile.com/c/RLgKWZ/934QJ), which were also reflected by generating huge modules in DiNiro. Therefore, we constructed the cell cluster (13,324 cells, 3635 genes), and created subnetworks based on more conservative parameters (p= 10**^-^**^6^, subsampling=4, subsampling size= 50%, occurrence threshold= 100%). The biggest module was regulated by HLF (hepatic leukemia factor) (Fig S2B S1 Table F). Recently, this TF has shown to be a key player in the pathogenesis of epilepsy [(18)](https://paperpile.com/c/RLgKWZ/pfoMd). In the network, it controls multiple genes significantly expressed in ASD or non-diseased L2/3 neurons (Fig S2B). Two genes (SSX2IP, HSP90AA1) upregulated in the ASD nuclei, were also reported in the study[(1)](https://paperpile.com/c/RLgKWZ/934QJ). As with microglia, we focused on the male vs. female nuclei (Fig S2C) (p= 10**^-^**^4^, subsampling=4, subsampling size= 70%, occurrence threshold= 100%). Out of 14 modules different between males and females, two modules also included DEGs reported differentially expressed in ASD vs non-diseased L2/3 neurons[(1)](https://paperpile.com/c/RLgKWZ/934QJ) (Fig S2D S1 Table G). Dysregulation of the GABA system is related to autism, [(19, 20)](https://paperpile.com/c/RLgKWZ/hc32b+nuQ8f) and in the female specific module, GABRB1 is present, a receptor subunit that mediates the fastest inhibitory synaptic transmission in the system (Fig S2D). This female module is regulated by RFC3, which has been detected in GABAergic neurons [(21)](https://paperpile.com/c/RLgKWZ/zQAlE). While in the male module, NLGN1 (neuroligin 1), a major component in excitatory glutamatergic neurons, is present, and it is also involved in synaptopathies in ASD [(22)](https://paperpile.com/c/RLgKWZ/0Nuzf). Several mutations in the gene have also been found in ASD patients [(23, 24)](https://paperpile.com/c/RLgKWZ/o7LDf+I0Ud1). NLGN1 is regulated by LUZP2 in the network module, which is also associated with both neurodegenerative and neuropsychiatric disease [(25)](https://paperpile.com/c/RLgKWZ/55oLK), and a variation in the gene has been reported in an ASD patient [(26)](https://paperpile.com/c/RLgKWZ/Zc2NT) (Fig S2D)

**Fig S2. L2/3 neuronal networks in autism spectrum disorder (ASD) vs non-diseased human brain tissue.** (A) tsne of snRNA-seq data of L2/3 neurons collected from 15 autism spectrum disorder (ASD) (red nuclei) and 16 control postmortem brain tissue (blue cells). (B) Selected DiNiro transcriptional gene regulatory network modules using significant cut off 10-6 for 4 subsamples with 50% subsampling size and 100% occurrence threshold. Green interaction lines are significant for ASD L2/3 neurons, red interaction lines for non-diseased L2/3 neurons. Detected significant L2/3 neuronal genes from the original study (Velmeshev et al. 2019) are highlighted in yellow. (C) tsne of snRNA-seq data of the L2/3 neuronal cluster as in (A). Nuclei from females colored in pink and nuclei from male in gray. (D) Selected DiNiro transcriptional gene regulatory network modules using significant cut off 0,0001 for 4 subsamples with 50% subsampling size and 100% occurrence threshold. Green interaction lines are significant for female L2/3 neurons, red interaction lines for male L2/3 neurons. Shared interactions have been removed (gray lines). Interactive network visualization S1 Tables F and G.

Altogether, with DiNiro we discover a more holistic connective picture of how reported individual genes related to ASD and brain pathology are connected. Additionally, DiNiro allows to reveal sex-specific differences in ASD patients. According to our network comparisons, changes in ASD patients are related to female specific excitatory- and male specific inhibitory neuronal pathways. This is also supported by a recent study, reporting a sex-different excitation-inhibition imbalance ratio in ASD [(27)](https://paperpile.com/c/RLgKWZ/qnaRW).

**2. Supplementary Tables**

The following tables state the different parameters used in each of the studies. in addition to DiNiro output links. Using the DiNiro output interface one can visualize the resulting networks as a whole or look into individual modules in addition to their gene set enrichment analysis results.

## **Network modeling of long-term antiviral CD8^+^ T cells: the key gene regulatory differences between cell fate of stemness vs terminal exhaustion**

**Table A.** Parameters and results of the study of long-term antiviral CD8^+^ T cells in the case of stem-like cell clusters (0_1) versus terminal exhausted cell clusters (1, 2).

| **parameters** | **value** |
| --- | --- |
| **significance cut-off** | 0.1 |
| **Subsamples** | 5 |
| **subsampling size** | 70% |
| **occurrence threshold** | 90% |
| **Study results** | |
| <https://exbio.wzw.tum.de/diniro/result2/?user=l6HM8PgolydijXW8zJfZjw28Ot55dfri> | |

**Table B.** Parameters and results of the study of long-term antiviral CD8^+^ T cells in the case of virus-specific CD8^+^ cells lacking BACH2 gene stem-like cell cluster (2) versus terminal exhausted cell clusters (0,1).

| **parameters** | **value** |
| --- | --- |
| **significance cut-off** | 0.1 |
| **Subsamples** | 5 |
| **subsampling size** | 70% |
| **occurrence threshold** | 90% |
| **Study results** | |
| <https://exbio.wzw.tum.de/diniro//result2/?user=YEq9PMXp18ZohllX9pjg5lRVaKMufqDU> | |

**Table C.** Parameters and results of the study of long-term antiviral CD8^+^ T stem-like cells in the case of Bach2gRNA versus controls.

| **parameters** | **value** |
| --- | --- |
| **significance cut-off** | 0.1 |
| **Subsamples** | 5 |
| **subsampling size** | 70% |
| **occurrence threshold** | 90% |
| **Study results** | |
| [https://exbio.wzw.tum.de/diniro//result2/?user=lARAOqgAYhKAmgDqA2mYvwJeAgo9SVkg](https://exbio.wzw.tum.de/diniro/result2/?user=lARAOqgAYhKAmgDqA2mYvwJeAgo9SVkg) | |

## **Network modeling of specific brain cell types predictive for clinical autism severity**

**Table D.** The study of microglia cells in the case of autism spectrum disorder (ASD) patients versus non-diseased controls.

| **parameters** | **value** |
| --- | --- |
| **significance cut-off** | 0.2 |
| **Subsamples** | 4 |
| **subsampling size** | 70% |
| **occurrence threshold** | 90% |
| **Study results** | |
| <https://exbio.wzw.tum.de/diniro//result2/?user=vFJiHg6PPD0RDNswYwO5MB5lDCkv4C6b> | |

**Table E.** The study of microglia cells in the case of male microglia versus female microglia.

| **parameters** | **value** |
| --- | --- |
| **significance cut-off** | 0.1 |
| **Subsamples** | 4 |
| **subsampling size** | 70% |
| **occurrence threshold** | 90% |
| **Study results** | |
| <https://exbio.wzw.tum.de/diniro//result2/?user=SInqqoOI1cotExodFq3miLNKBcIBHrP4> | |

**Table F.** The study of L2/3 neuronal cells in the case of autism spectrum disorder (ASD) patients versus non-diseased controls.

| **parameters** | **value** |
| --- | --- |
| **significance cut-off** | 10**^-^**^6^ |
| **Subsamples** | 4 |
| **subsampling size** | 50% |
| **occurrence threshold** | 100% |
| **Study results** | |
| <https://exbio.wzw.tum.de/diniro//result2/?user=qAWeJ6MbVrnKAQqXRUwhqiiL1mzCfxIL> | |

**Table G.** The study of L2/3 neuronal cells in the case of male L2/3 neuronal versus female L2/3 neuronal.

| **parameters** | **value** |
| --- | --- |
| **significance cut-off** | 10**^-^**^4^ |
| **Subsamples** | 4 |
| **subsampling size** | 70% |
| **occurrence threshold** | 100% |
| **Study results** | |
| <https://exbio.wzw.tum.de/diniro//result2/?user=VKHxXpXzMw6fCT5vJNsrFIOjcFGKAdii> | |

##

## **Network modeling of SARS-Cov 2 infected cells**

**Table H.** The study of Airway Basal Stem Cells (ABSCs) cells in the case of SARS-CoV-2 infected cells versus non-infected cells.

| <https://exbio.wzw.tum.de/diniro//result2/?user=b6AmdlAlE7cBHeLfecrAbE1ohMgHaN7E> | |
| --- | --- |
| **parameters** | **value** |
| **significance cut-off** | 10**^-^**^28^ |
| **Subsamples** | 4 |
| **subsampling size** | 50% |
| **occurrence threshold** | 90% |
| **Study results** | |
| <https://exbio.wzw.tum.de/diniro//result2/?user=b6AmdlAlE7cBHeLfecrAbE1ohMgHaN7E> | |

# **3. Supplementary Notes: The effects of parameter settings on the analysis**

DiNiro default parameters are Number of subsamples=4, Sub-sampling size (%)=70, Occurrence threshold (%)=70, Significance cutoff=0.05. These parameters have been found to elicit the desired effect behind the sub-sampling and permutation tests used for statistical significance assessment, offering a starting point before fine-tuning. We recommend that the user go through the effect of this parameter (detailed below) and refer to the Method section before choosing the appropriate value for these parameters based on their data and experiment design.

### **Significance cutoff:**

This cutoff is based on the p-value calculated as explained in the numerical methods. The lower the p-value is, the more significant are the results.

We advise the user to use a non-conservative approach and start with a p-value ≥ 0.05 then fine tune this parameter accordingly.

**Species selection:**

During the co-expression step the correlations are only computed between the transcription factors (TFs) and genes. For this purpose it is mandatory to select one of the species (Human or Mouse), in order for the tool to use the right TFs files. Selecting the wrong file or no intersection between the uploaded TFs will result in an empty output.

**Number of subsamples:**

Here, the user specifies the number of random sub-samples to extract from each sample. A large number will result in increasing the computational time and getting more conservative results. Users must also consider the degree of heterogeneity among the cells in the sample when selecting this number. We recommend that the user choose values in the range of 4-8.

**Sub-sampling size (%):**

The percentage of cells from the total number of cells of the sample to be included in each random subsample. In case of the sample containing only one cell type, we recommend values in the range of 40%-70%, in case of sample formed from multiple clusters the range becomes 50%-80% (if none of the clusters forms more the 50% of the total cells in the sample) to avoid confusing cell type heterogeneity with noise coming from technical issues of single cell.

**Occurrence threshold (%):**

To reduce noise, gene regulatory networks are computed multiple times and gene interactions are filtered based on their occurrence threshold (in percentage).

See **Methods** for a detailed explanation.

# **References**

[1. Velmeshev,D., Schirmer,L., Jung,D., Haeussler,M., Perez,Y., Mayer,S., Bhaduri,A., Goyal,N., Rowitch,D.H. and Kriegstein,A.R. (2019) Single-cell genomics identifies cell type–specific molecular changes in autism. *Science*, **364**, 685–689.](http://paperpile.com/b/RLgKWZ/934QJ)

[2. Fernø,J., Raeder,M.B., Vik-Mo,A.O., Skrede,S., Glambek,M., Tronstad,K.-J., Breilid,H., Løvlie,R., Berge,R.K., Stansberg,C., *et al.* (2005) Antipsychotic drugs activate SREBP-regulated expression of lipid biosynthetic genes in cultured human glioma cells: a novel mechanism of action? *Pharmacogenomics J.*, **5**, 298–304.](http://paperpile.com/b/RLgKWZ/dtBDT)

[3. Fernø,J., Skrede,S., Vik-Mo,A.O., Håvik,B. and Steen,V.M. (2006) Drug-induced activation of SREBP-controlled lipogenic gene expression in CNS-related cell lines: marked differences between various antipsychotic drugs. *BMC Neurosci.*, **7**, 69.](http://paperpile.com/b/RLgKWZ/JYhRQ)

[4. Sampieri,L., Di Giusto,P. and Alvarez,C. (2019) CREB3 Transcription Factors: ER-Golgi Stress Transducers as Hubs for Cellular Homeostasis. *Front Cell Dev Biol*, **7**, 123.](http://paperpile.com/b/RLgKWZ/AbSqq)

[5. Teh,D.B.L., Prasad,A., Jiang,W., Ariffin,M.Z., Khanna,S., Belorkar,A., Wong,L., Liu,X. and All,A.H. (2017) Transcriptome Analysis Reveals Neuroprotective aspects of Human Reactive Astrocytes induced by Interleukin 1β. *Sci. Rep.*, **7**, 13988.](http://paperpile.com/b/RLgKWZ/c7Byg)

[6. Howard,A. and Rogers,A.N. (2014) Role of translation initiation factor 4G in lifespan regulation and age-related health. *Ageing Res. Rev.*, **13**, 115–124.](http://paperpile.com/b/RLgKWZ/UZhFP)

[7. Salem,S., Salem,D. and Gros,P. (2020) Role of IRF8 in immune cells functions, protection against infections, and susceptibility to inflammatory diseases. *Hum. Genet.*, **139**, 707–721.](http://paperpile.com/b/RLgKWZ/ScxNQ)

[8. Masuda,T., Tsuda,M., Yoshinaga,R., Tozaki-Saitoh,H., Ozato,K., Tamura,T. and Inoue,K. (2012) IRF8 is a critical transcription factor for transforming microglia into a reactive phenotype. *Cell Rep.*, **1**, 334–340.](http://paperpile.com/b/RLgKWZ/C7zIL)

[9. Rustenhoven,J., Smith,A.M., Smyth,L.C., Jansson,D., Scotter,E.L., Swanson,M.E.V., Aalderink,M., Coppieters,N., Narayan,P., Handley,R., *et al.* (2018) PU.1 regulates Alzheimer’s disease-associated genes in primary human microglia. *Mol. Neurodegener.*, **13**, 44.](http://paperpile.com/b/RLgKWZ/TPr1E)

[10. International Multiple Sclerosis Genetics Consortium (2019) Multiple sclerosis genomic map implicates peripheral immune cells and microglia in susceptibility. *Science*, **365**.](http://paperpile.com/b/RLgKWZ/jjpaH)

[11. Hargis,K.E. and Blalock,E.M. (2017) Transcriptional signatures of brain aging and Alzheimer’s disease: What are our rodent models telling us? *Behavioural Brain Research*, **322**, 311–328.](http://paperpile.com/b/RLgKWZ/94bj2)

[12. Ma,W., Cojocaru,R., Gotoh,N., Gieser,L., Villasmil,R., Cogliati,T., Swaroop,A. and Wong,W.T. (2013) Gene expression changes in aging retinal microglia: relationship to microglial support functions and regulation of activation. *Neurobiology of Aging*, **34**, 2310–2321.](http://paperpile.com/b/RLgKWZ/ngA8x)

[13. Suzuki,K., Sugihara,G., Ouchi,Y., Nakamura,K., Futatsubashi,M., Takebayashi,K., Yoshihara,Y., Omata,K., Matsumoto,K., Tsuchiya,K.J., *et al.* (2013) Microglial activation in young adults with autism spectrum disorder. *JAMA Psychiatry*, **70**, 49–58.](http://paperpile.com/b/RLgKWZ/yiGAN)

[14. Gupta,S., Ellis,S.E., Ashar,F.N., Moes,A., Bader,J.S., Zhan,J., West,A.B. and Arking,D.E. (2014) Transcriptome analysis reveals dysregulation of innate immune response genes and neuronal activity-dependent genes in autism. *Nat. Commun.*, **5**, 5748.](http://paperpile.com/b/RLgKWZ/nf74T)

[15. Werling,D.M. and Geschwind,D.H. (2013) Sex differences in autism spectrum disorders. *Curr. Opin. Neurol.*, **26**, 146–153.](http://paperpile.com/b/RLgKWZ/UCfZZ)

[16. Yanguas-Casás,N. (2020) Physiological sex differences in microglia and their relevance in neurological disorders. *Neuroimmunology and Neuroinflammation*,](http://paperpile.com/b/RLgKWZ/Oq7da) [10.20517/2347-8659.2019.31](http://dx.doi.org/10.20517/2347-8659.2019.31)[.](http://paperpile.com/b/RLgKWZ/Oq7da)

[17. Guneykaya,D., Ivanov,A., Hernandez,D.P., Haage,V., Wojtas,B., Meyer,N., Maricos,M., Jordan,P., Buonfiglioli,A., Gielniewski,B., *et al.* (2018) Transcriptional and Translational Differences of Microglia from Male and Female Brains. *Cell Rep.*, **24**, 2773–2783.e6.](http://paperpile.com/b/RLgKWZ/XpDot)

[18. Rambousek,L., Gschwind,T., Lafourcade,C., Paterna,J.-C., Dib,L., Fritschy,J.-M. and Fontana,A. (2020) Aberrant expression of PAR bZIP transcription factors is associated with epileptogenesis, focus on hepatic leukemia factor. *Sci. Rep.*, **10**, 3760.](http://paperpile.com/b/RLgKWZ/pfoMd)

[19. Fatemi,S.H., Reutiman,T.J., Folsom,T.D. and Thuras,P.D. (2009) GABA(A) receptor downregulation in brains of subjects with autism. *J. Autism Dev. Disord.*, **39**, 223–230.](http://paperpile.com/b/RLgKWZ/hc32b)

[20. Coghlan,S., Horder,J., Inkster,B., Andreina Mendez,M., Murphy,D.G. and Nutt,D.J. (2012) GABA system dysfunction in autism and related disorders: From synapse to symptoms. *Neuroscience & Biobehavioral Reviews*, **36**, 2044–2055.](http://paperpile.com/b/RLgKWZ/nuQ8f)

[21. Wu,S., Esumi,S., Watanabe,K., Chen,J., Nakamura,K.C., Nakamura,K., Kometani,K., Minato,N., Yanagawa,Y., Akashi,K., *et al.* (2011) Tangential migration and proliferation of intermediate progenitors of GABAergic neurons in the mouse telencephalon. *Development*, **138**, 2499–2509.](http://paperpile.com/b/RLgKWZ/zQAlE)

[22. Nakanishi,M., Nomura,J., Ji,X., Tamada,K., Arai,T., Takahashi,E., Bućan,M. and Takumi,T. (2017) Functional significance of rare neuroligin 1 variants found in autism. *PLoS Genet.*, **13**, e1006940.](http://paperpile.com/b/RLgKWZ/0Nuzf)

[23. Glessner,J.T., Wang,K., Cai,G., Korvatska,O., Kim,C.E., Wood,S., Zhang,H., Estes,A., Brune,C.W., Bradfield,J.P., *et al.* (2009) Autism genome-wide copy number variation reveals ubiquitin and neuronal genes. *Nature*, **459**, 569–573.](http://paperpile.com/b/RLgKWZ/o7LDf)

[24. Trobiani,L., Meringolo,M., Diamanti,T., Bourne,Y., Marchot,P., Martella,G., Dini,L., Pisani,A., De Jaco,A. and Bonsi,P. (2020) The neuroligins and the synaptic pathway in Autism Spectrum Disorder. *Neurosci. Biobehav. Rev.*, **119**, 37–51.](http://paperpile.com/b/RLgKWZ/I0Ud1)

[25. Stepanov,V., Vagaitseva,K., Bocharova,A., Marusin,A., Markova,V., Minaycheva,L. and Makeeva,O. (2018) Analysis of Association of Genetic Markers in the LUZP2 and FBXO40 Genes with the Normal Variability in Cognitive Performance in the Elderly. *Int. J. Alzheimers. Dis.*, **2018**, 2686045.](http://paperpile.com/b/RLgKWZ/55oLK)

[26. Walker,S. and Scherer,S.W. (2013) Identification of candidate intergenic risk loci in autism spectrum disorder. *BMC Genomics*, **14**, 499.](http://paperpile.com/b/RLgKWZ/Zc2NT)

[27. Trakoshis,S., Martínez-Cañada,P., Rocchi,F., Canella,C., You,W., Chakrabarti,B., Ruigrok,A.N., Bullmore,E.T., Suckling,J., Markicevic,M., *et al.* (2020) Intrinsic excitation-inhibition imbalance affects medial prefrontal cortex differently in autistic men versus women. *Elife*, **9**.](http://paperpile.com/b/RLgKWZ/qnaRW)
